# Lupus with initial mesenteric vasculitis

**DOI:** 10.2478/rir-2023-0015

**Published:** 2023-07-22

**Authors:** Jing-Jing Xie, Gui-Chen Ling, Yu-Bao Jiang, Jian-Yong Zhang

**Affiliations:** The Department of Rheumatology, the Fourth Clinical Medical College of Guangzhou University of Chinese Medicine, Shenzhen 518033, Guangdong Province, China; The Department of Rheumatology, Shenzhen Traditional Chinese Medicine Hospital, Shenzhen 518033, Guangdong Province, China

A 39-year-old woman was admitted because of abdominal pain, nausea, and vomiting after meals for 2 days. She also had fecal incontinence, urination frequency, and urgency for 4 days. She had no specific past medical history. Physical examination revealed diffuse abdominal distension, tenderness below the sternum, and rebound pain without rigidity. Laboratory tests showed mild anemia, positive homogeneous pattern antinuclear antibody (ANA) with a titer of 1:320, she was also positive for anti-U1-RNP antibodies, anti-double-stranded DNA antibodies, anti-nucleosome antibodies, anti-SSA antibodies, anti-RO-52 antibodies, and antihistone antibodies, with decreased complements levels. Her D-dimer was 2745 μg/L. Enhanced computed tomography of the abdomen revealed diffuse circumferential wall thickening with submucosal edema of the entire small bowel, accompanied by ascites. “Fence-like” changes of mesenteric vessels with dilatation of bowel loops, thickened bowel walls, and the “double-halo” sign or “target” sign were observed (Figure 1). The diagnosis of systemic lupus erythematosus (SLE) and mesenteric vasculitis was made. She was treated with intravenous methylprednisolone 200 mg per day for 5 days, followed by methylprednisolone 60 mg everyday combined with rituximab 500 mg every two weeks twice. She was also treated with gastric and intestinal decompression. Her symptoms disappeared.

**Figure 1:**
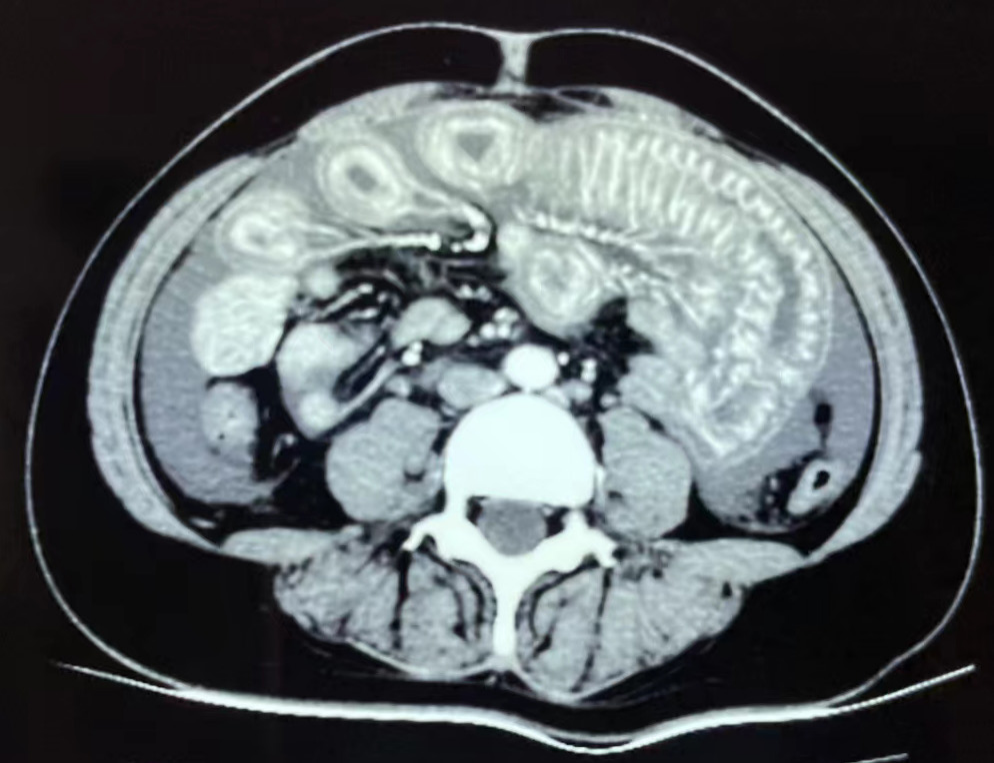
A computed tomography (CT) scan showed changes in the mesenteric vessels, dilated bowel loops, thickened bowel walls, and the “double-halo” or “target” sign before the start of treatment.

Lupus mesenteric vasculitis (LMV) is an uncommon manifestation of SLE that can be difficult to diagnose.^[1]^ Patients initially presenting with mesenteric vasculitis have an even higher misdiagnosis rate. Abdominal computed tomography (CT) is an important imaging examination for early detection of LMV, including “fence-like” abnormalities, dilation of the bowel loops, thickening of the bowel wall, and “double-halo” sign.^[2]^ Early diagnosis of LMV and immediate intervention can prevent potentially fatal complications and unnecessary surgical intervention.

## Acknowledgements

We would like to thank the guidance and advice by Jiu-Liang Zhao at Chinese Academy of Medical Sciences and Peking Union Medical College.
